# ZnO Nano-Particles Production Intensification by Means of a Spinning Disk Reactor

**DOI:** 10.3390/nano10071321

**Published:** 2020-07-05

**Authors:** Marco Stoller, Javier Miguel Ochando-Pulido

**Affiliations:** 1 Department of Chemical Materials Environmental Engineering, Sapienza University of Rome, 00136 Rome, Italy; 2 Department of Chemical Engineering, Granada University, 18071 Granada, Spain; jmochandop@ugr.es

**Keywords:** process intensification, spinning disk reactor, nano zinc oxide

## Abstract

Zinc Oxide is widely used in many industrial sectors, ranging from photocatalysis, rubber, ceramic, medicine, and pigment, to food and cream additive. The global market is estimated to be USD 3600M yearly, with a global production of 10 Mt. In novel applications, size and shape may sensibly increase the efficiency and a new nano-ZnO market is taking the lead (USD 2000M yearly with a capacity of 1 Mt and an expected Compound Annual Growth Rate of 20%/year). The aim of this work was to investigate the possibility of producing zinc oxide nanoparticles by means of a spinning disk reactor (SDR). A lab-scale spinning disk reactor, previously used to produce other nanomaterials such as hydroxyapatite or titania, has been investigated with the aim of producing needle-shaped zinc oxide nanoparticles. At nanoscale and with this shape, the zinc oxide particles exhibit their greatest photoactivity and active area, both increasing the efficiency of photocatalysis and ultraviolet (UV) absorbance. Working at different operating conditions, such as at different disk rotational velocity, inlet distance from the disk center, initial concentration of Zn precursor and base solution, and inlet reagent solution flowrate, in certain conditions, a unimodal size distribution and an average dimension of approximately 56 nm was obtained. The spinning disk reactor permits a continuous production of nanoparticles with a capacity of 57 kg/d, adopting an initial Zn-precursor concentration of 0.5 M and a total inlet flowrate of 1 L/min. Product size appears to be controllable, and a lower average dimension (47 nm), adopting an initial Zn-precursor concentration of 0.02 M and a total inlet flow-rate of 0.1 L/min, can be obtained, scarifying productivity (0.23 kg/d). Ultimately, the spinning disk reactor qualifies as a process-intensified equipment for targeted zinc oxide nanoparticle production in shape in size.

## 1. Introduction

Nowadays, nanoparticle production has increased in a notable way due to its widespread number of applications in different sectors—such as electronic and energy storage [1,2,3,4], industrial catalysis [5,6], pharmaceutical and biomedical [3,7,8,9], food [10,11,12], civil and waste re-use [13,14,15,16], and the environment [13,17,18,19,20,21,22,23]. The production of nanoparticles requires particular operating conditions and equipment, in particular the achievement of micro-mixing conditions [24,25] to avoid rapid aggregation phenomena [26,27], or the use of complexing agents to reduce surface electrostatic attraction among nanoparticles, such as carboxy-methyl-cellulose, alginate, xanthan and other organic compounds [28,29]. Metallic and metal oxide nanoparticle synthesis has been widely studied, and many articles can be found in the literature [30]. Among metal oxide nanoparticles, ZnO attracted the interest of focused research due to its extraordinary electronic, optical, mechanical, magnetic and chemical properties, that are significantly different from those of the bulk counterpart: high chemical stability, high electrochemical coupling coefficient, a broad range of radiation absorption, paramagnetic nature and high photostability [31]. In particular, the lack of a center of symmetry in wurtzite, combined with large electromechanical coupling, results in strong piezoelectric and pyroelectric properties, and the consequent use of ZnO in mechanical actuators and piezoelectric sensors [32]. ZnO is a wide band-gap (3.37 eV) compound semiconductor, which is suitable for various kind of applications, such as ultraviolet (UV) lasers, power generators, solar cells, etc., whereas ZnO in powder is widely used as an additive to numerous materials and products, including ceramics, glass, cement, rubber, and in skin lotion [33]. ZnO has been produced by various methods, using vapor deposition or chemical precipitation methods [34]. The latter method, prepared in its liquid phase, is advantageous for the production of nanoparticle suspensions, which avoids, among other things, the dispersion of its material in the environment. Since the activity of ZnO is so high, it can result in greater amounts of toxicity and requires inhibition by the addition of surfactants [35,36]. Chemical precipitation was mainly investigated using classical lab-scale glassware, and to the author’s knowledge, no other particular equipment (either at lab or pilot-scale) has been employed in the past. The use of a spinning disk reactor (SDR) for inorganic and metal oxide nanoparticles production has been studied in the last two decades by a small number of research teams, and less than 100 articles on this subject can be found in the literature [37,38,39,40,41,42,43,44]. However, the adoption of such equipment may allow for the rapid scaling-up of classical batch productions of nanoparticles, achieving the advantages of a continuous production process (waste reduction, larger reproducibility and production rate, reduced manufacturing cost, higher and consistent product quality, etc. [45]), and the production of size and shape controlled nanoparticles from a bottom-up approach. 

The present work reports the investigation of the nano-ZnO production intensification process by the use of a lab-scale SDR, analyzing the influence of the main operative parameters on the shape, average dimension, and size distribution of the produced particles.

## 2. Materials and Methods 

All of the reagents were purchased from Carlo Erba (Milan, Italy) and were of analytical grade, whereas all of the solutions were prepared with deionized water. The following reagents have been adopted in the experiments: Zinc Sulphate Heptahydrate (ZnSO_4_·7H_2_O, purity > 99.95%, M = 287.49 g/mol) was used as a Zn(II) precursor, whereas KOH (purity > 99.00%) was used as the base, for the formation and precipitation of Zn(OH)_2_, according to the Equation (1):

(1) $${\mathrm{ZnSO}}_{4}+2\mathrm{KOH}\to \mathrm{Zn}{\left(\mathrm{OH}\right)}_{2}+K_{2}{\mathrm{SO}}_{4}$$

The molar ratio used in the production experiments was fixed as Zn(II)/OH = 0.25 mol/mol, according to the optimal results obtained in preliminary experiments (data not showed). 

**Figure 1:**
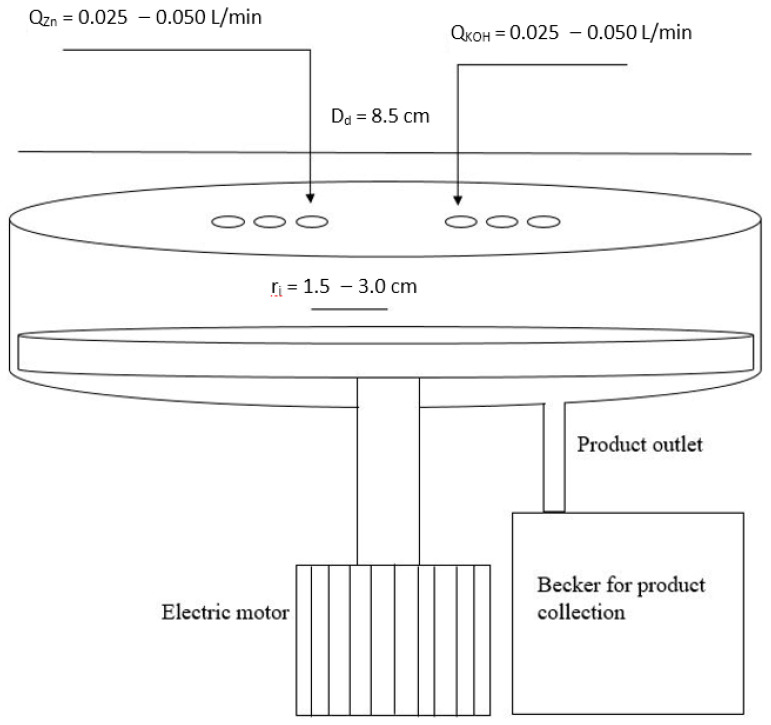
Spinning disk reactor (SDR) schematization.

The SDR used in this work is schematically reported in Figure 1, to allow insight into the main part of this equipment, which would not be possible by displaying a photo (since it is a boxed equipment).

The equipment consists of an inner disk made of Teflon, with a 4.25 cm radius inside an external cylinder (stator part). The feed streams are injected onto the disk surface, which rotates by means of an electric motor. The distance from the injection point to the disk center can be varied, hereafter called *r_i_* [cm], from 3.0 cm down to 1.5 cm at 0.5 cm steps, whereas the rotational velocity *ω* [rpm] can be varied from 0 rpm up to 1400 rpm. The reagent solutions have been injected, maintaining constancy at 25 °C—the temperature of the water batch. The internal diameter of the feed stream tubes was 3 mm.

A total of 25 experimental runs were performed. The production experiments were performed varying the following parameters as reported in brackets: Zn(II) initial concentration (0.02–2.00 M, and, as a consequence, KOH molar concentration varied in the range 0.08–8.00 M), reagent solution inlet flowrates (*Q_Zn_* and *Q_KOH_* [L/min], 0.025–0.500 L/min), *ω* (400–1400 rpm) and *r_i_* (1.5–3.0 cm). Referring to the data reported in Table 1, the pH of the produced solution was in the range 12.51–12.62 in the first 16 runs. Then, at higher Zn(II), i.e., KOH molarity, the pH increased: in runs 17–19 it was in the range 13.0–13.5, and in the remaining runs, it reached 13.7–14.0. At the end of each test, the Zn(OH)_2_ precipitated and was separated from the liquid by centrifugation (12,000 rpm, 15 min). The solid residue was washed three times with ethanol and was placed in the oven for 24 h at 105 °C. The obtained powder was then characterized and the size distribution and average diameter, *d* [nm], were measured by the Dynamic Light Scattering method, using a Zetasizer Nano ZS (Malvern Panalytical, Westborough, MA, USA), and the pH was measured using a Crison pH-meter (Barcellona, Spain). The pH value during measurements was almost the same for all of the samples, and equal to 6.0. The yield of ZnO was measured as the ratio between ZnO produced mass and Zn(II) initial mass. The most convincing productions were characterized using a SEM-EDS (Zeiss, Oberkochen, Germany). 

Initially, the Zn(II) and KOH molar concentrations were set equal to 0.02 and 0.08 M, and the maximum rotational velocity (1400 rpm) value was fixed, according to the optimal results obtained on metallic iron nanoparticles in a previous work [34], varying both the reagent solution flowrate (0.025, 0.050, 0.075 and 0.100 L/min) and *r_i_* (1.5, 2.0, 2.5 and 3.0 cm). Once the optimal reagent flowrate and *r_i_* values were found, all of the operating parameters were fixed, varying *ω* (200, 400, 800, 1200 rpm). Therefore, once the *ω* optimal value was found, the Zn(II) and KOH molar concentrations were varied (0.05/0.20, 0.10/0.40, 0.20/0.80, 0.5/2.0 and 1.0/4.0 M, respectively). Finally, an additional 4 runs were carried out, fixing the reagent flowrates at 0.5 L/min and varying the Zn(II) and KOH molar concentrations (0.5/2.0, 0.75/3.0, 1.0/4.0 and 2.0/8.0 M, respectively). Each experiment was carried out in duplicate, and the average values of the obtained results were reported.

The productivity *P* [kg/d] was calculated as the multiplication of the total flowrate (converted in l/d), initial Zn concentration (converted in kg/L), and yield of ZnO.

## 3. Results

In this section, the experimental results of the 25 runs will be reported. A summary table (Table 1) of the investigated operating conditions is reported.

### 3.1. Influence of Reagents Flowrate and Reagent Injection Point Position 

**Figure 2:**
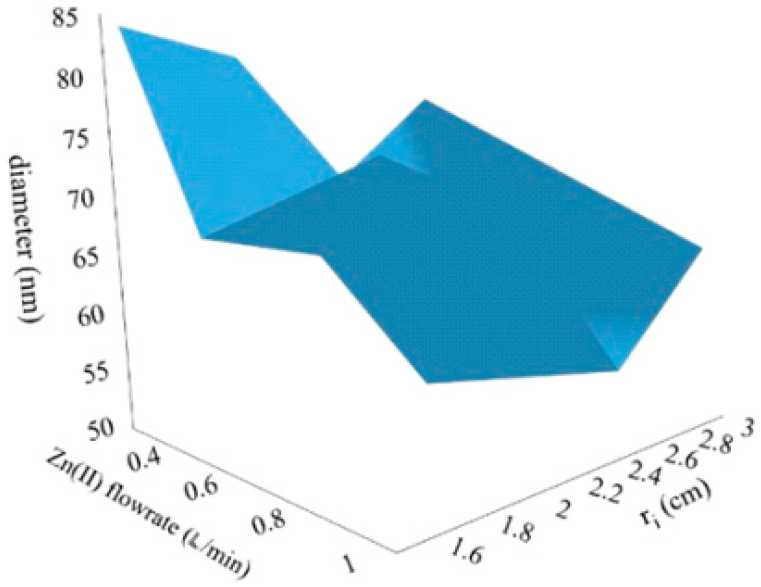
3D graph of average nZnO diameter (*d* [nm]) in function of varied reagent solution flowrate and *r_i_*.

Figure 2 repots the average diameter of nZnO as a function of different *Q_Zn_* (i.e., *Q_KOH_*) and *r_i_* values.

Table 2 reports the detail of the results obtained in the first 12 runs.

### 3.2. Influence of Disk Rotational Velocity and Reagent’s Concentration

**Figure 3:**
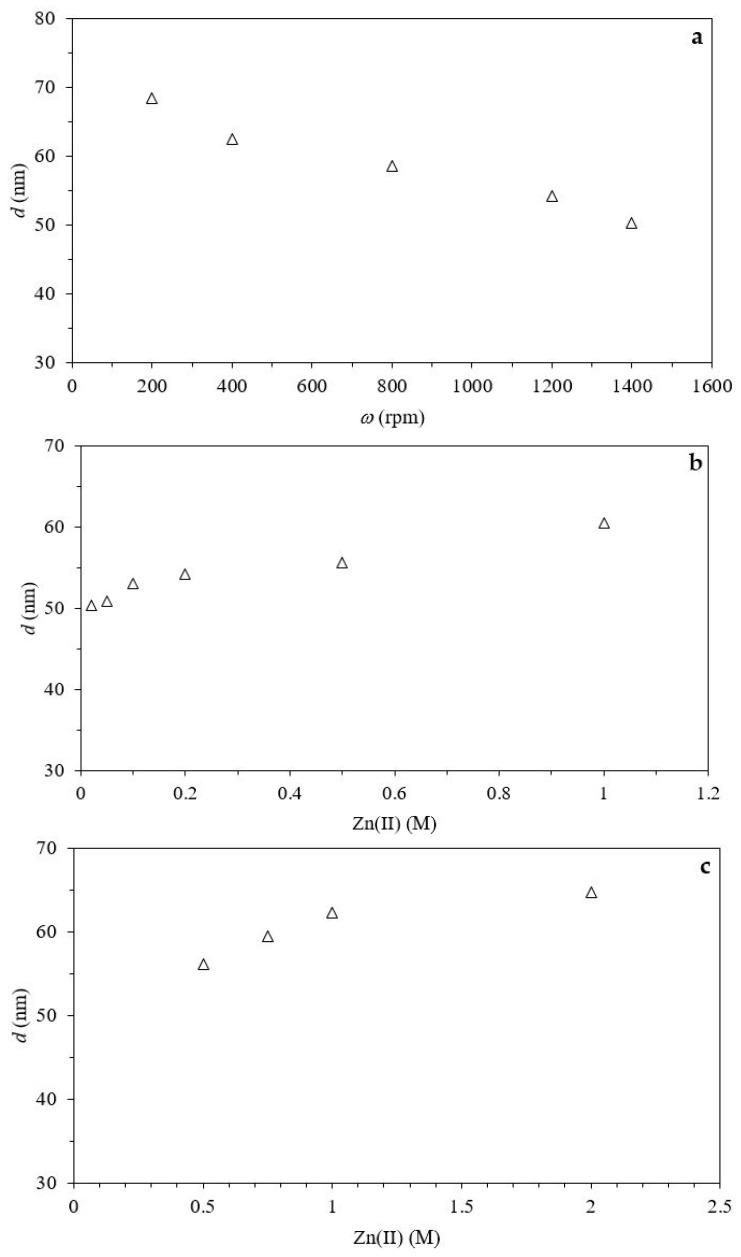
Influence of *ω* (**a**) and Zn(II)/KOH initial concentration (**b**) and (**c**) on measured *d*.

Figure 3 reports the average diameter of nZnO measured at different *ω* (a) and Zn(II)/KOH molar concentrations at 0.05 L/min (b) and 0.5 L/min (c).

Table 3 reports the detail of the results obtained in the remaining 13 runs.

### 3.3. Morphology of Obtained nZnO Particles

**Figure 4:**
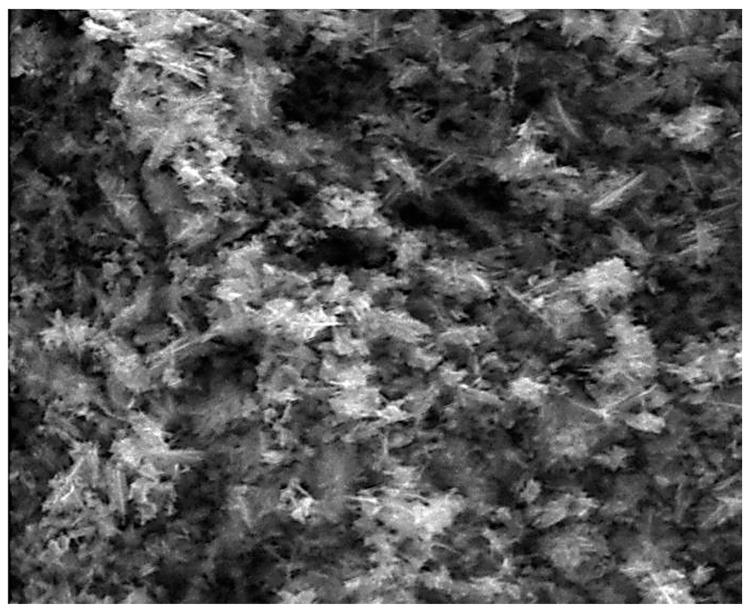

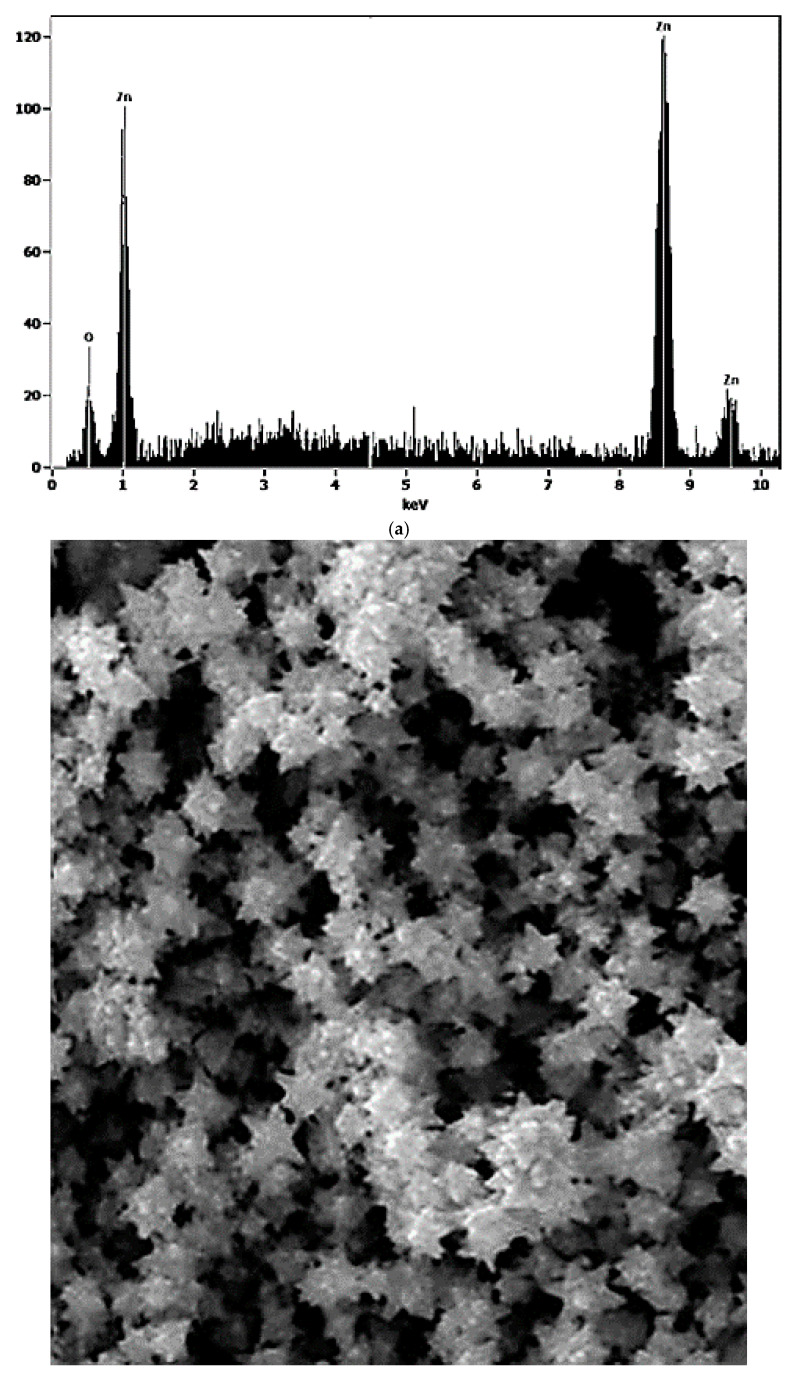

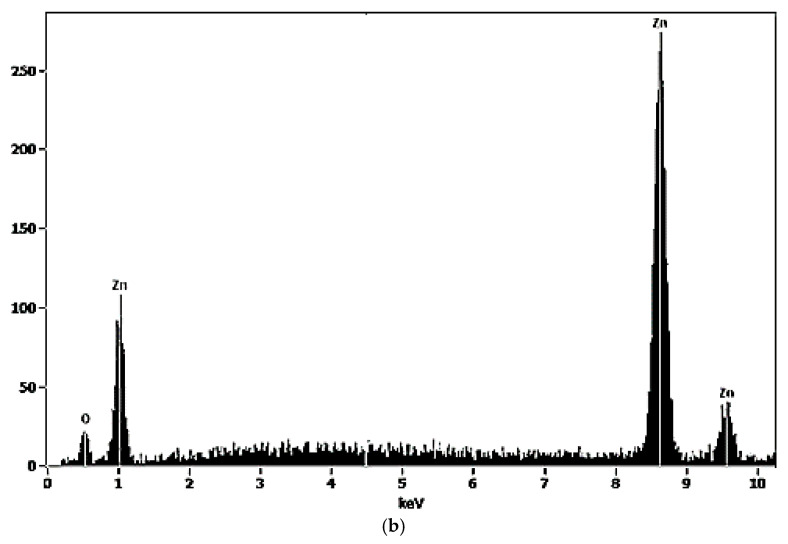
Structure of nZNO obtained in run 23 (**a**), and 8 (**b**) (EHT = 20.00 kV, Mag = 160.00kx, WD = 9.5 mm).

Figure 4 displays the SEM and EDX results obtained of the nZnO produced in runs: 23 (a, *Q_Zn_* = 0.05 L/min, *ω* = 1400 rpm, *r_i_* = 2.5 cm and Zn(II) = 0.02 M) and 8 (c, *Q_Zn_* = 0.5 L/min, *ω* = 1400 rpm, *r_i_* = 2.5 cm and Zn(II) = 0.5 M). The first one was selected as it exhibited the highest productivity still characterized by a unimodal particle size distribution; the second because it performed at the same operating conditions, but at 1/10 of the reagent concentrations compared with run 23. 

## 4. Discussion

**Figure 5:**
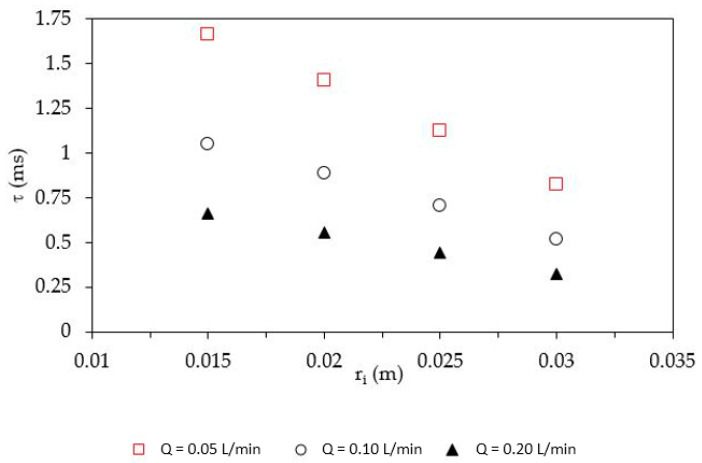
*r_i_* influence on residence time.

The influence of *r_i_* on the nZnO particles and total flowrate on the rotating disk was clearly visible in Figure 2. Indeed, the optimal values were 2.5 cm for *r_i_* and 0.05 L/min for *Q_Zn_*, i.e., 0.1 L/min as the total flowrate. These two parameters strongly influenced the fluid dynamic conditions established in the rotating liquid film, which was generated onto the surface of the disk [46]. The residence time *τ* [s] on the disk can be calculated as [37]:

(2) $$\tau ={\left(\frac{81\pi^{2}\upsilon }{16\omega^{2}Q^{2}}\right)}^{1/3}\left(r_{d}^{4/3}-r_{i}^{4/3}\right)$$

where *Q* is the total inlet flowrate [m^3^/s], *ν* is the kinematic viscosity [m^2^/s], assumed equal to that of water being the diluted solution, and *r_d_* [m] is the disk radius. Figure 5 summarizes the influence of *r_i_* on residence time. 

**Figure 6:**
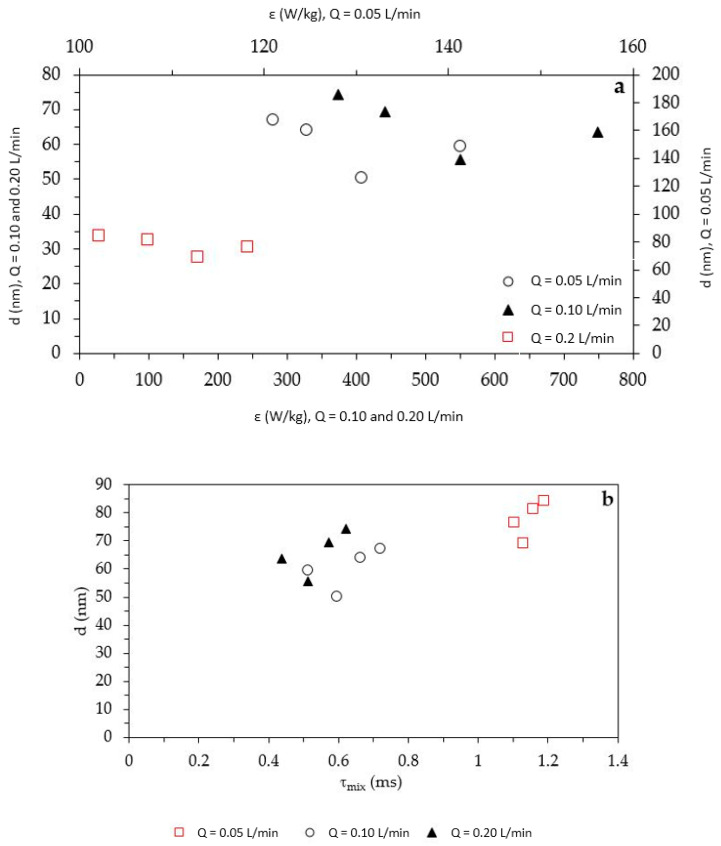
*d* trend with respect to *ε* (**a**) and *τ_mix_* (**b**).

The increase of *r_i_* and *Q* caused a decrease in the reagents residence time on the rotating disk surface; therefore, *τ* being too low, or *Q* too large, may hinder completion of the reaction. Usually, these kind of precipitation reactions performed by the SDR are very rapid [38], with induction times lower than 1–1.5 ms. Therefore, the influence of the analyzed parameters on the reaction yield was quite limited within the investigated range. Indeed, at fixed *r_i_*, an increase in *Q* caused a yield reduction of approximately 1–3% (see Table 2), whereas when maintaining *Q* as constant, an increase in *r_i_* led to a yield reduction of 1–2%. These parameters also influenced the average dimension of the obtained nanoparticles, mainly due to the different specific power values dispersed in the rotating liquid film *ε* [W/kg] and mixing time *τ_mix_* [s], as already observed in previous works [10,24]:

(3) $$\varepsilon =\frac{1}{2\tau }\left[{\left(\omega^{2}r^{2}+{\bar{v_{r}}}^{2}\right)}_{\mathrm{out}}-{\left(\omega^{2}r^{2}+{\bar{v_{r}}}^{2}\right)}_{\mathrm{feed}}\right]$$

(4) $$\tau_{\mathrm{mix}}=12{\left(\frac{\upsilon }{\varepsilon }\right)}^{0.5}$$

where *v_r_* [m/s] is the average radial velocity calculated according to a simplified centrifugal model [35]. Figure 6 displays the influence of *ε* (a) and of *τ_mix_* (b) on the nZnO particles mean diameter.

The mean particle’s diameter trend with respect to *ε* and *τ_mix_* was, of course, analogous, as the latter parameter depends on the former, and showed that the same minimum occurred for *r_i_* = 2.5 cm, at all *Q* values. The order of magnitude of *ε* and *τ_mix_* were in line with those obtained in a previous study, working at similar operative conditions (*Q* = 0.1–0.2 L/min) [34]. It has already been demonstrated that when the mixing time of the SDR is in the order of 0.1–1 ms, the obtained particles can reach dimensions lower than 100 nm, as obtained in the present study [35]. 

The subsequent runs were performed by fixing the *Q* to 0.1 L/min and the *r_i_* to 2.5 cm, according to the lower mean diameter obtained. The influence of the Zn(II) precursor molar concentration on *d* was quite limited, compared with that of *ω* (see Figure 3). This can be explained by considering that when the SDR is used, the average size of the produced nanoparticles is mainly influenced by *ε* and of *τ_mix_* (i.e., by *ω* and fluid dynamic conditions), whereas the initial reagents concentration can influence d only to some extent, as reported in previous works, where classical synthesis pathway and equipment were also used. 

The results obtained by the SDR are well comparable with those reported in literature by Liu and Zeng [47], Gao et al. [48] and Wirunmongkol et al. [49]. In this work, the mean hydrodynamic diameter of the obtained particles was always in the range of 50–80 nm, with a lower OH^−^/Zn(II) molar ratio in comparison with those reported in the aforementioned studies, where the most commonly used ratio value was 10/1. In detail, in the first study, ZnO nanorods of an effective diameter of approximately 50 nm, were obtained by means of hydrothermal synthesis at 180 °C for 20 h, using ethylenediamine (EDA) at an EDA/Zn(II) molar ratio of 50/1, in addition to the adoption of a Zn(II)/OH^−^ molar ratio of 20/1. Therefore, the synthesis required a higher energy demand and a larger quantity of reagents to obtain a homogeneous rod-like structure for the product, if compared with the SDR equipment. Regarding the second mentioned study [45], the authors adopted a OH^−^/Zn(II) molar ratio equal to 10/1 and produced ZnO nanorods with an average diameter of approximately 100 nm, assembled into sphere-like structures, by means of hydrothermal synthesis at 95 °C for 5 h, using hexamethylenetetramine (HMT) at a HMT/Zn(II) molar ratio equal to 1/1. Finally, the last study [46] reported ZnO nano-rods assembled in flower-structures, similar to those reported in the present study (Figure 4a,b), with a mean diameter in the range of 30–80 nm, using hydrothermal synthesis (60 °C for 6 h) and adopting a OH^−^/Zn(II) molar ratio equal to 10/1. The main influence of the OH^−^ concentration was on the ZnO nanoparticles morphology variation: at a lower OH^−^/Zn(II) molar ratio (2/1), they were of spherical shape, and at a higher molar ratio (10/1–20/1), they became rod/wire-shaped, when the classical hydrothermal synthesis was adopted. Another important consideration reported in the aforementioned studies was the influence of the initial Zn(II) concentration: an increase in the precursor molar concentration usually led to an increase in the average diameter and a change in the morphology, causing an increase in the mean diameter and the occurrence of rod/wire-shaped particles. Analogous considerations were made in another study, where a continuous hydrothermal synthesis by supercritical water was reported [50]. Analogous results have been reported in the present study, where the flower-shaped particles obtained at a Zn(II) concentration of 0.5 M (Figure 4b), they changed their morphology to wire/rod-shaped when the Zn(II) concentration decreased down to 0.02 M (Figure 4a). The initial reagents concentration also influenced the shape of the Particle Size Distribution (PSD): indeed, bimodal PSD occurred when the Zn(II) molar rate was larger than 0.5 M—both at low and high inlet flowrates. This can be explained by considering that at higher reagent concentration and at fixed or lower liquid film volume values (as at higher inlet flowrate), the volume concentration of the ions quickly increased, leading to more possibilities for nucleation reactions, but also crystal growth and aggregation phenomena [51,52,53].

Overall, the production of the chemical precipitation of nano ZnO by means of a spinning disk reactor, does not appear to require particular precautions to work continuously for a long period of time. Scaling in the reactor was absent after the experimental campaign, and all of the particles were suspended in the liquid, which did not evaporate at the adopted temperature values. 

## 5. Conclusions

In conclusion, the SDR demonstrated itself to be a suitable equipment for the intensification of nano-ZnO particles production, as it was able to obtain good performances in terms of average size (approximately 50 nm), high yield (>97%), and unimodal particle size distribution. The same could be achieved by adopting a larger inlet flowrate and initial Zn(II) precursor concentration, in order to increase the production rate. Indeed, the last four runs of the reported experimental set showed that it was possible to keep the modal particle size below 60 nm using a Zn(II) molar concentration <1 M. In these conditions, productivity values were higher than 50 kg/d, making the overall production process useful for industrial application. Higher inlet flowrates were not suggested, as these operating conditions led to a sensible increase in the average dimension of particles and the size distribution, changing from a unimodal to a bimodal one. However, further studies should be performed to investigate the possibility of achieving larger production rates (up to 1000 kg/d), without exiting the range of the desired product’s characteristics.

## Figures and Tables

**Table 1:** Experimental conditions adopted in the 25 runs.

ID	Q_Zn_ (L/min)	Q_KOH_ (L/min)	Zn(II) (M)	KOH (M)	*ω* (rpm)	*r_i_* (cm)
1	25	25	0.02	0.08	1400	1.5
2	50	50	0.02	0.08	1400	1.5
3	100	100	0.02	0.08	1400	1.5
4	25	25	0.02	0.08	1400	2
5	50	50	0.02	0.08	1400	2
6	100	100	0.02	0.08	1400	2
7	25	25	0.02	0.08	1400	2.5
8	50	50	0.02	0.08	1400	2.5
9	100	100	0.02	0.08	1400	2.5
10	25	25	0.02	0.08	1400	3
11	50	50	0.02	0.08	1400	3
12	100	100	0.02	0.08	1400	3
13	50	50	0.02	0.08	200	2.5
14	50	50	0.02	0.08	400	2.5
15	50	50	0.02	0.08	800	2.5
16	50	50	0.02	0.08	1200	2.5
17	50	50	0.2	0.8	1400	2.5
18	50	50	0.1	0.4	1400	2.5
19	50	50	0.05	0.2	1400	2.5
20	50	50	0.5	2	1400	2.5
21	50	50	1	4	1400	2.5
22	500	500	0.5	2	1400	2.5
23	500	500	0.75	3	1400	2.5
24	500	500	1	4	1400	2.5
25	500	500	2	8	1400	2.5

**Table 2:** Results obtained in the first 12 runs.

ID	Q_Zn_ (L/min)	Q_KOH_ (L/min)	Zn(II) (M)	KOH (M)	*w* (rpm)	*r_i_* (cm)	*d* (nm)	Yield	*P* (kg/d)	PSD ^1^
1	25	25	0.02	0.08	1400	1.5	84.2	0.961	0.113	Unimodal
2	50	50	0.02	0.08	1400	1.5	67.1	0.956	0.224	Unimodal
3	100	100	0.02	0.08	1400	1.5	74.3	0.949	0.445	Unimodal
4	25	25	0.02	0.08	1400	2	81.3	0.980	0.115	Unimodal
5	50	50	0.02	0.08	1400	2	64.1	0.972	0.228	Unimodal
6	100	100	0.02	0.08	1400	2	69.5	0.966	0.453	Unimodal
7	25	25	0.02	0.08	1400	2.5	69.1	0.982	0.116	Unimodal
8	50	50	0.02	0.08	1400	2.5	50.3	0.979	0.229	Unimodal
9	100	100	0.02	0.08	1400	2.5	55.7	0.968	0.454	Unimodal
10	25	25	0.02	0.08	1400	3	76.4	0.961	0.116	Unimodal
11	50	50	0.02	0.08	1400	3	59.4	0.956	0.230	Unimodal
12	100	100	0.02	0.08	1400	3	63.7	0.952	0.456	Unimodal

^1^ Particle Size Distribution.

**Table 3:** Results obtained in the remaining 13 runs.

ID	Q_Zn_ (L/min)	Q_KOH_ (L/min)	Zn(II) (M)	KOH (M)	*w* (rpm)	*r_i_* (cm)	*d* (nm)	Yield	P (kg/d)	PSD ^1^
13	50	50	0.02	0.08	200	2.5	68.4	0.979	0.23	Bimodal
14	50	50	0.02	0.08	400	2.5	62.4	0.979	0.23	Bimodal
15	50	50	0.02	0.08	800	2.5	58.5	0.985	0.23	Bimodal
16	50	50	0.02	0.08	1200	2.5	54.1	0.992	0.23	Unimodal
17	50	50	0.2	0.8	1400	2.5	54.2	0.992	2.32	Unimodal
18	50	50	0.1	0.4	1400	2.5	53.1	0.991	1.16	Unimodal
19	50	50	0.05	0.2	1400	2.5	50.9	0.993	0.58	Unimodal
20	50	50	0.5	2	1400	2.5	55.6	0.991	5.81	Unimodal
21	50	50	1	4	1400	2.5	60.4	0.991	11.60	Bimodal
22	500	500	0.5	2	1400	2.5	56.1	0.983	57.60	Unimodal
23	500	500	0.75	3	1400	2.5	59.4	0.981	86.22	Unimodal
24	500	500	1	4	1400	2.5	62.3	0.982	115.08	Bimodal
25	500	500	2	8	1400	2.5	64.7	0.983	230.39	Bimodal

^1^ Particle Size Distribution.
